# The effect of interval time of transcatheter arterial chemoembolization with drug-eluting beads on the efficacy and safety of unresectable hepatocellular carcinoma: a case-control study

**DOI:** 10.3389/fonc.2025.1679167

**Published:** 2025-10-20

**Authors:** Guoyu Deng, Huaqing Zhang, Haotian Xue, Kaizhong Zheng, Chang Zhao

**Affiliations:** Interventional Department, Affiliated Cancer Hospital of Guangxi Medical University, Nanning, Guangxi, China

**Keywords:** transcatheter hepatic arterial chemoembolization, drug-eluting beads, hepatocellular carcinoma, interval time, carcinoma

## Abstract

**Purpose:**

To investigate the effect of treatment interval on the efficacy and safety of transcatheter arterial chemoembolization with drug-eluting beads (DEB-TACE) in patients with unresectable hepatocellular carcinoma (HCC).

**Methods:**

Retrospective analysis of clinical data of HCC patients admitted to our hospital from December 2015 to December 2023. Kaplan Meier method was used to calculate survival rate, survival curve was plotted, log rank test was used for univariate analysis, and Cox regression model was used to analyze independent prognostic factors. Select cutoff values based on OS using X-tile software for grouping, and compare the impact of time intervals on OS and adverse reactions.

**Results:**

The median OS of the entire group was 26 months, and the 1-, 3-, and 5-year survival rates were 85.6%, 48.3%, and 41.8%, respectively. Multi factor analysis shows that, BCLC, The occurrence of splenomegaly, targeted therapy, and TACE interval are independent prognostic factors for overall survival. The analysis of treatment interval grouping showed that the cut-off value of TACE time interval was 4 weeks. The group with TACE interval>4 weeks (long interval group) showed better survival benefits than the group with TACE interval<4 weeks (short interval group) (mOS: 47 *vs* 34 months, P<0.001). The sub group analysis results showed that in the sub group analysis of ECOG grade 0 patients, no distant metastasis, and Child Pugh A patients, the long interval group had longer OS than the short interval group. One week after the second postoperative follow-up and comparison of laboratory indicators between the two groups, the differences in lactate dehydrogenase (LDH) and platelets between the two groups were significant (P<0.05). No serious treatment-related complications were observed in any of the patients.

**Conclusion:**

DEB-TACE performed at intervals longer than 4 weeks has a better prognosis for HCC than DEB-TACE performed within 4 weeks without increasing adverse reactions.

## 1 Introduction

Hepatocellular carcinoma (HCC) continues to increase in incidence rate and mortality in China and other countries with high incidence of liver disease (1–3). Most patients in China are already in the middle or late stages of the illness when identified, limiting the use of standard surgical treatment (4).

Transcatheter arterial chemoembolization (TACE) has become one of the standard chemotherapeutic choices for patients with non-resectable HCC (5). Drug-eluting beads (DEB) are used to provide sustained targeted release of cytotoxic drugs while blocking tumor vasculature. The existing literature points out that DEB-TACE can be used as a safe, feasible and effective palliative treatment for patients with unresectable or recurrent HCC (6). In addition, compared with traditional TACE, DEB-TACE showed a lower incidence of postoperative pain while increasing the risk of hepatic artery and biliary damage (7).Existing literature indicates that compared with traditional TACE, DEB-TACE increases the risk of hepatic artery and bile duct damage while showing a lower incidence of postoperative pain (7). Although DEB-TACE has achieved significant clinical efficacy in the treatment of HCC, there is currently no consensus on the specific impact of different DEB-TACE treatment intervals on the treatment efficacy and safety (such as treatment-related adverse events).

Therefore, this study aims to explore the effects of different treatment intervals on the efficacy and safety of DEB-TACE. Through case-control studies, we hope to provide clearer guidance for clinical practice and help determine the optimal interval for TACE treatment, thereby improving the treatment effect of patients, and reducing adverse reactions.

## 2 Materials and methods

### 2.1 Clinical data

Collect clinical data of patients diagnosed with advanced HCC in our hospital from December 2015 to December 2023. Inclusion criteria: (1) patients diagnosed with HCC by imaging examination (CT or MRI) and whose lesions could not be removed by surgery; (2) age range: 18–80 years old; (3) patients whose liver function evaluation met the conditions for TACE treatment. Exclusion criteria: (1) patients with other types of liver cancer or metastatic liver cancer; (2) patients with severe liver failure or other serious systemic diseases; (3) patients with obvious bleeding and coagulation disorders or severe infections; (4) pregnant or lactating women or patients of childbearing age who were planning family planning; (5) patients who were unable to cooperate with follow-up. 436 HCC patients were divided into a short interval group (<4 weeks, 114 cases) and a long interval group (>4 weeks, 322 cases) based on the interval of TACE treatment. The hospital ethics committee approved this study with ethics approval number KY2025594.

### 2.2 Treatment methods

The Seldinger technique was used to insert the catheter through the right femoral artery. The location, size and blood supply of the tumor were evaluated by angiography. After the angiography results were determined, the microcatheter was used to further superselectively insert the tumor blood supply artery. The group was slowly injected with a pre-prepared chemotherapy drug solution, including CalliSpheres drug-loaded microspheres (Hengrui Jiali Biomedicine, specifications 100-300 μm), loaded with pirarubicin for injection (Shenzhen Wanle Pharmaceutical, specifications 20 mg/branch) 50 mg and fully mixed. After standing, ioversol was injected at a ratio of 1: 1, and the drug solution was 15–20 mL. Observe the blood supply of arterial blood flow stagnation, 5 minutes after repeated angiography to confirm, pull out the catheter, withdraw the arterial sheath, puncture site pressure bandage; the patient was instructed to brake the affected limb for 6 hours and lie flat for 12 hours. The monitoring of vital signs and puncture sites was strengthened, and symptomatic treatment such as analgesia, stomach protection, antiemetic, liver protection and support was given. After 4 weeks of follow-up, if there was a new lesion or an increase in the primary lesion, the above treatment could be repeated once after contraindications were excluded.

### 2.3 Observation indicators

Collected basic information of patients. Compare overall survival (OS), and treatment-related adverse events were evaluated. OS is defined as the period from the date of pathological diagnosis of HCC to the date of death of the patient for any reason, or the date of the last follow-up, measured in months. All patients will receive outpatient or inpatient follow-up at 1, 2, 3 months after surgery, and every 3 months thereafter, during which clinical symptoms, tumor progression rate, liver reserve, and postoperative recovery will be evaluated, and enhanced CT or MRI examinations will be performed. If the primary tumor diameter is significantly enlarged or new lesions are found during follow-up, the patient will receive another TACE treatment or ablation treatment according to the situation, and may be combined with targeted drug therapy. All patients follow a unified collection protocol for imaging examinations before and after treatment. The timing, imaging scanning methods, and parameter settings of CT/MRI examinations have been strictly standardized to ensure comparability and consistency of data. In addition, imaging examinations are conducted by experienced imaging experts to minimize subjective interpretation errors as much as possible.

### 2.4 Statistical analysis

Perform statistical analysis using SPSS 23.0 software. Count data is presented in the form of frequency (percentage/%), and analysis of variance is used for inter group comparisons; For metric data that conforms to a normal distribution, mean ± standard deviation is used for description, and t-test or analysis of variance is used. OS is represented by M (P25, P75), survival curves are analyzed using Kaplan Meier survival analysis, inter group survival curves are compared using Log Rank test, and prognostic factors are analyzed using Cox regression. P<0.05 indicates a statistically significant difference.

## 3 Results

### 3.1 Univariate analysis of factors influencing overall prognosis

The results of the single factor analysis are shown in **Table 1**. The results show that ECOG(P<0.001).BCLC(P<0.001). Splenomegaly (P = 0.025), hepatitis B (P<0.001), targeted therapy (P = 0.033), TACE time interval (P<0.001) were correlated with patients’ OS (P<0.05).

**Table 1:** General information and survival time of HCC patients.

Factor		Total, n%	OS	
			mOS	P
gender	male	378	42	0.070
	female	58	49	
age	≤60	362	43	0.518
	>60	74	37	
ECOG	0	361	44	<0.001
	1	67	39	
	3	8	3	
Family history	have	362	43	0.219
	none	74	47	
Drinking history	have	276	37	0.074
	none	160	46	
BCLC	Phase 0	19	58	<0.001
	Phase A	126	53	
	Phase B	73	42	
	Phase C	209	twenty two	
	D phase	9	2	
Tumor size	<3cm	37	48	0.223
	3-5cm	303	43	
	>5cm	96	twenty two	
Vascular invasion	none	233	42	0.080
	have	158	40	
	Above the trunk	45	47	
Location	right	277	43	0.718
	Left	62	31	
	about	78	46	
	The junction of the upper right anterior lobe and the left medial lobe	13	50	
	Right lobe of liver and caudate lobe of liver	6	46	
number	1	300	42	0.072
	2	56	34	
	3	17	29	
	4	9	5	
	Multiple	9	49	
	Multiple	45	47	
Distant metastasis	none	396	51	0.050
	have	40	41	
Cirrhosis	have	322	41	0.128
	none	114	49	
Splenomegaly	have	245	35	0.025
	none	191	44	
ascites	have	66	41	0.128
	none	370	51	
Esophageal varices	have	104	42	0.993
	none	332	44	
Hepatitis B	have	379	39	<0.001
	none	57	57	
Hepatitis C	have	19	42	0.061
	none	417	53	
Child-Pugh	A	360	41	0.545
	B	73	46	
	C	3	51	
Targeted therapy	have	245	35	0.025
	none	191	44	
TACE time interval	<4 weeks	138	34	<0.001
	>4 weeks	298	47	

### 3.2 Analysis of factors influencing OS

In order to further analyze the influencing factors of patients’ OS, we used ECOG, BCLC, splenomegaly, hepatitis B, targeted treatment and TACE time interval as covariates to conduct a multifactor Cox proportional risk analysis. See **Table 2** for the assignment. The results show that BCLC, Whether splenomegaly occurs, targeted therapy, and TACE interval are independent influencing factors of OS (P<0.05), as shown in **Table 3**.

**Table 2:** Assignment.

Project	Assignment
ECOG	1 = Level 0; 2 = Level 1; 3 = Level 3
BCLC	1 = Period 0; 2 = Period A; 3 = Period B; 4 = Period C; 5 = Period D
Splenomegaly	1 = yes; 0 = no
Hepatitis B	1 = yes; 0 = no
Targeted therapy	1 = yes; 0 = no
TACE time interval	1=>4 weeks; 0=<4 weeks

**Table 3:** COX regression analysis of factors affecting OS.

Project	B	SE	Wald χ 2	P	HR (95% CI)
ECOG			2.027	0.363	
ECOG(1)	-0.158	0.206	0.583	0.445	0.854 (0.570-1.280)
ECOG(2)	0.571	0.493	1.342	0.247	1.770 (0.674-4.651)
BCLC			47.930	<0.001	
BCLC(1)	0.316	0.402	0.621	0.431	1.372 (0.625-3.015)
BCLC(2)	0.760	0.409	3.449	0.063	2.138 (0.959-4.768)
BCLC(3)	1.208	0.393	9.462	0.002	3.348 (1.550-7.229)
BCLC(4)	2.708	0.626	18.699	< 0.001	15.000 (4.396-51.186)
Splenomegaly	0.316	0.140	5.108	0.024	1.371 (1.043-1.803)
Hepatitis B	-0.081	0.182	0.199	0.655	0.922 (0.645-1.317)
Targeted therapy	1.235	0.521	5.613	0.018	3.437 (1.238-9.545)
TACE time interval	0.877	0.315	7.761	0.005	2.403 (1.297-4.452)

### 3.3 Comparison of the impact of TACE interval on patient OS

The last follow-up was on December 30, 2024. There was no statistically significant difference in general information between the long interval (>4 weeks) group and the short interval (<4 weeks) group.

**Figure 1:**
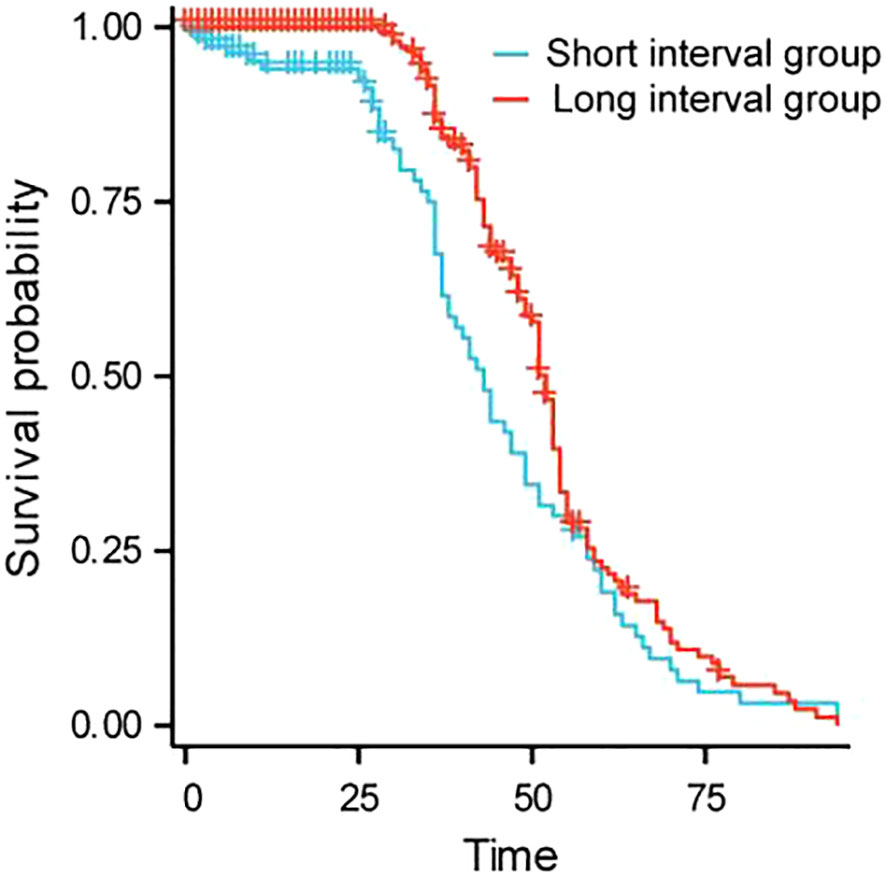
Survival curves of patients.

The median survival time for the short interval group is 34 (confidence interval: 23-41) months, while the median survival time for the long interval group is 47 (confidence interval: 43-51) months; There was a statistically significant difference in OS between the two groups (HR = 1.585, P<0.001). The results showed that the survival rate of the short interval group within 5 years was lower than that of the long interval group. Refer to **Figure 1**.

### 3.4 Subgroup analysis

**Figure 2:**
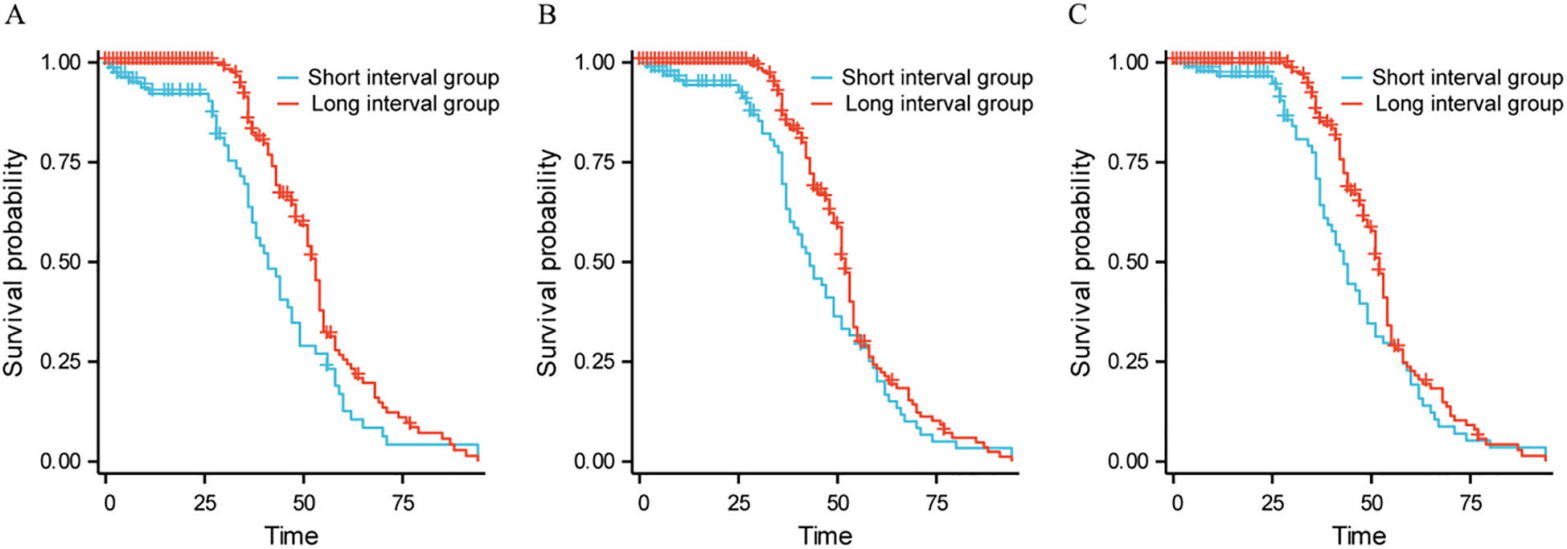
Survival curves of subgroup analysis. Subgroup analysis of patients with ECOG grade 0; **(B)**. Subgroup analysis of patients without distant metastasis; **(C)**. Subgroup analysis of patients with Child-Pugh **(A)**.

In the subgroup analysis of patients with ECOG grade 0, patients without distant metastasis, and Child-Pugh A, the long-interval group had longer OS (χ 1 2 = 8.117, HR 1 = 0.630, P 1 = 0.004; χ 2 2 = 4.179, HR 2 = 0.738, P 2 = 0.041; χ 3 2 = 3.982, HR 3 = 0.736, P 3 = 0.046), see **Figure 2**.

### 3.5 Comparison of postoperative laboratory parameters

All patients were re-examined 1 week after the second surgery, and the laboratory test results were compared, see **Table 4**. The differences in lactate dehydrogenase (LDH) and platelets were significant (P < 0.05).

**Table 4:** Comparison of postoperative laboratory indicators.

Project	Short interval group	Long interval group	T	P
Total bilirubin (μ mol/L)	16.15 ± 8.59	16.98 ± 13.70	0.602	0.548
Direct bilirubin (μ mol/L)	6.59 ± 4.06	6.92 ± 6.70	0.504	0.615
Total protein (g/L)	71.06 ± 7.98	69.94 ± 7.44	1.355	0.176
Albumin (g/L)	39.75 ± 4.82	39.06 ± 5.34	1.216	0.225
Alanine aminotransferase (ALT) (U/L)	64.32 ± 80.51	58.84 ± 59.40	0.767	0.444
Aspartate aminotransferase AST (U/L)	77.24 ± 69.13	73.23 ± 74.21	0.504	0.615
Lactate dehydrogenase LDH (U/L)	343.88 ± 476.42	253.82 ± 138.97	3.051	0.002
Glutamyl transpeptidase GGT (U/L)	153.92 ± 172.40	141.94 ± 164.14	0.661	0.509
Total bile acid (μ mol/L)	20.01 ± 31.96	16.89 ± 20.21	1.199	0.231
Serum creatinine (μ mol/L)	77.74 ± 18.40	78.90 ± 17.37	0.604	0.546
Coagulation time (s)	12.94 ± 1.78	13.15 ± 1.83	1.085	0.278
INR	1.00 ± 0.13	1.01 ± 0.13	0.992	0.322
AFP (ng/ml)	1204.86 ± 2250.99	1236.65 ± 2197.32	0.132	0.895
White blood cell count (×10^9^/L)	7.33 ± 2.51	6.89 ± 3.89	1.124	0.262
Platelet count (×10^9^/L)	216.34 ± 77.61	180.22 ± 83.06	4.057	<0.001

### 3.6 Security analysis

No serious complications related to treatment were observed in all patients. See **Table 5**. There was no significant difference between two groups (P > 0.05). All complications were resolved by symptomatic treatment during hospitalization, and there was no treatment-related death.

**Table 5:** Postoperative complications of the two groups of patients.

Grouping	Number of cases	Liver abscess	Bleeding	Infect	χ2	P
Short interval group	114	1	2	2	1.792	0.617
Long interval group	322	3	1	3		

## 4 Discussions

HCC makes up about 75% to 80% of all cases of primary liver cancer (8). It is worth noting that the incidence of HCC is increasing year by year, especially in developing countries in Asia, where its incidence exceeds half of the global total. Although a variety of risk factors are known to predict the occurrence of HCC, the mortality rate associated with it is still rising. In China, less than 12.5% of people with liver cancer survive for five years (9). DEB-TACE is widely used in palliative treatment or interventional treatment of patients with unresectable HCC, playing an important role in maintaining drug concentration in the tumor and sustained drug release. Research has demonstrated that DEB-TACE works better than C-TACE for treating intrahepatic cholangiocarcinoma and HCC (10, 11). Compared with cTACE, DEB-TACE has better therapeutic effect, higher survival rate and lower incidence of adverse events (12). In addition, DEB-TACE can improve patient tolerance, reduce hospital stay, and have a more lasting target tumor response (13). However, so far, studies on the optimal DEB-TACE treatment interval have not reached a consensus. The results of univariate analysis showed that patients’ ECOG, BCLC, splenomegaly, hepatitis B, targeted therapy and TACE interval were the influencing factors of OS (P<0.05).

Further multivariate Cox proportional hazards regression analysis revealed that, BCLC, Whether splenomegaly occurs, targeted therapy, and TACE interval are independent influencing factors of OS (P<0.05). BCLC staging is the most commonly used HCC staging system in Western countries, aimed at dividing patients into five different prognostic stages and allocating treatment based on these stages (14). In our study, BCLC staging was significantly correlated with overall survival (OS), with patients in BCLC stage 0 and BCLC stage A having significantly better survival than those in BCLC stage B and C. This result is consistent with previous studies (15). BCLC staging can not only evaluate the progression of tumors, but also reflect the degree of liver function damage, thus it has a strong predictive effect on the prognosis of patients. Early stage liver cancer patients usually receive better treatment outcomes, while late stage patients may have limited treatment effects due to liver dysfunction and tumor expansion. Splenomegaly, as one of the common complications of hepatocellular carcinoma, is usually associated with portal hypertension in the liver (16). Splenomegaly caused by splenic hyperfunction may lead to the destruction and reduction of blood cells, causing anemia in patients and ultimately affecting their survival (17). Some studies suggest that high spleen volume is a predictive indicator of low survival rate in HCC patients, therefore a combination of splenectomy and liver resection should be adopted for such patients (18). Targeted therapy has become one of the important methods for treating hepatocellular carcinoma in recent years (19, 20). In this study, the application of targeted therapy significantly improved overall survival (OS), suggesting that targeted therapy may improve patient survival by inhibiting tumor angiogenesis and enhancing chemotherapy efficacy. Targeted drugs such as sorafenib (21) and regorafenib (22) have been proven to have good therapeutic effects on advanced liver cancer patients and have been approved as systemic treatment regimens for HCC (23). In addition, immune checkpoint inhibitors have also shown good survival benefits (24). In addition, the time interval of TACE has important clinical significance in the treatment of liver cancer. Our multivariate Cox regression analysis found that the time interval of TACE was independently correlated with OS, providing a basis for optimizing treatment plans. Usually, the treatment interval of TACE is closely related to the reduction of tumor size, recovery of liver function, and patient tolerance. Therefore, how to plan the interval time of TACE treatment reasonably to balance the treatment effect and patient tolerance is the key to improving the treatment effect.

Further analysis showed that the median survival time in the short interval group was 34 months, while the median survival time in the long interval group was 47 months; There was a statistically significant difference in OS between the two groups (HR = 1.585, P<0.001). The results showed that the survival rate of the short interval group within 5 years was lower than that of the long interval group. Shorter treatment intervals may lead to excessive burden on liver function, which in turn can cause tumor progression. Subgroup analysis results showed that in the subgroup analysis of ECOG grade 0 patients, patients without distant metastasis and Child-Pugh A patients, the long interval group had a longer OS than the short interval group. The ECOG score is a tool to assess the patient’s physical strength and functional status. ECOG grade 0 patients usually have no physical dysfunction, good physiological status and strong tolerance. Prolonging the treatment interval may help reduce the cumulative toxicity of drugs and the burden on patients, provide more recovery time, reduce side effects, and improve immune function, thereby improving the quality of life and prolonging OS. For patients without distant metastasis, a long treatment interval helps local tumor control and allows patients more time to recover, thereby improving long-term survival. For patients without distant metastasis, the focus of the treatment strategy is to maximize local control and reduce systemic side effects, and the long interval just provides a good balance for this treatment goal. Child-Pugh A indicates that the patient has good liver function and can withstand more treatment load. Such patients have good liver function reserves and can benefit from a longer treatment interval to reduce liver damage after embolization. Earlier research has demonstrated that TACE can enhance the outlook for patients with intermediate-stage HCC (25, 26). The findings of this study revealed that in patients classified as Child-Pugh A, those in the long-interval group lived significantly longer than those in the short-interval group. This result further verifies the importance of liver function status in TACE treatment, suggesting that in patients with good liver function, a long-interval treatment strategy may help prolong the patient’s OS.

Lactate dehydrogenase (LDH) is an enzyme that helps change lactate into pyruvate during the process of cellular metabolism. This enzyme is found in many different tissues throughout the body, particularly in important organs like the liver, heart, muscles, and kidneys (27). Research has indicated that high LDH levels are linked to a worse outlook for cancer patients (28). One week after the surgery, the rise in LDH levels in the short interval group might indicate greater cell damage and tissue stress after the operation when compared to the long interval group. Moreover, LDH may serve as a possible biomarker to forecast the recovery and complication chances for patients with HCC after surgery (29). Platelets are important cell components in the blood and are involved in blood coagulation and hemostasis (30). High platelet counts indicate poor prognosis for HCC patients (31). In this study, there was a significant difference in platelet counts between the two groups of patients, and long-term interval DEB-TACE treatment was more beneficial for the prognosis of patients.

This study has certain limitations. Firstly, as a retrospective case-control study, its research design may be affected by incomplete and biased retrospective data. Although we have adjusted for potential confounding factors through multivariate Cox regression analysis, we cannot completely rule out the influence of selection bias. Secondly, the small sample size may limit the broad applicability of the conclusions. Future prospective studies can further validate the results of this study and explore the optimal treatment plan for TACE at different time intervals.

Medical image segmentation, as a core step in medical image analysis, plays a crucial role in defining lesion areas, assisting clinical diagnosis decision-making, and developing personalized treatment plans (32–35). The highly vascularized nature of the liver makes it particularly important to accurately evaluate the hemodynamic parameters of its related vascular system, such as blood flow velocity, intravascular pressure, and wall shear stress. This has significant implications for optimizing treatment options and precise implementation of interventional therapy (36–38). Therefore, future research can further combine advanced image segmentation techniques to explore the clinical efficacy and mechanism of action of TACE, thereby improving the scientificity and objectivity of efficacy evaluation.

In conclusion, DEB-TACE with a long time interval has a better OS and no serious adverse reactions. Tumor location, presence of splenomegaly, ascites, and esophageal varices at the gastric fundus are independent factors affecting the patient’s postoperative OS.

## Data Availability

The raw data supporting the conclusions of this article will be made available by the authors, without undue reservation.
